# Dr. Watt’s Address

**Published:** 1853-07

**Authors:** 


### DENTAL REGISTER OF THE WEST.

Vol. VI. JULY, 1853, No. 4.

### Original Communications.

### DR. WATT'S ADDRESS

*Before the Mississippi Valley Association of Dental Surgeons, held in Cincinnati, Feb. 18 and 19, 1853.*

Mr. President and Gentlemen:—At the last meeting of our Association, it was my lot to be appointed to read an essay at the present session. No subject was assigned, and I have had difficulty in selecting one. Being young in the profession, we do not feel like boring our brethren with ideas stale to them.

The Dental Profession, as regards utility, influence, and respectability, occupies a prominent position in society. This appears especially true when compared with its former standing. The attainments made in the last few years, far surpass those of all former periods. From a low position, it has risen to rank and respectability; from small beginnings it has grown great; once in the clutches of empiricism, now it is in the hands of science; formerly it professed but little, and failed to fulfil its promises; now it promises much, and surpasses the hopes of its patrons.

With all these attainments, however, we must hope and believe that Dental science is still in its infancy. Each year adds important truths and enlists new talents.

Shall we rest satisfied with our present attainments, or shall we still advance? I hope that all enlisted in our cause feel determined to go forward. If our profession is to be elevated to a position higher than it occupies at present, it must be done by the exertions of its own members, for the non-professional are all otherwise occupied, each one acting out his own part in life's drama.

A few suggestions relative to the advancement of the Dental profession, without much regard to system, is all that I aim at in this essay.

By observing the past, we learn the importance of scientific research, and the necessity of special attention to the sciences pertaining to Dental Surgery. As long as Dental science was confined to Medical schools, but trifling attainments were made. The barber, the blacksmith, and the physician were joint practitioners of Dental Surgery, and, in general, had equal reputation for skill and dexterity. But when scientific men gave Dental science their especial and exclusive attention, as might well be expected, important results were soon developed.

The formation of Dental colleges, the adoption of a thorough course of collegiate studies, and the high qualifications requisite for graduation, have done more, we presume, to elevate the profession, than any other class of agencies. These colleges, at first, labored under various difficulties, and encountered many discouragements. These arose, in part, from the nature of the undertaking. It is difficult to plan and execute important achievements. Many mistakes and partial failures may be expected, and much patience and perseverance are required in bringing them to perfection.

Man is a selfish animal, (the poets to the contrary, notwithstanding), and our profession has its proportion of self-love. In organizing the faculties of the colleges, that selfish spirit could not, in all cases, be gratified by promotion; hence, jealousy stepped in among us, and our greatest foes were those of our own household.

Our colleges triumphed. They supplied the wants and gained the confidence of the enlightened portion of the profession; and the intelligent public looks to them as the means of furnishing efficient practitioners to supplant the empiric. But, as soon as they are found occupying prominent positions, opposition, from various sources, assails them. Many members of the profession, and some of high standing, propose to annihilate them, and to substitute "Dental chairs" in Medical schools.

In general, I believe, this kind of opposition originates with those who are in the vicinity of Medical schools, and who fancy they would stand a fair chance for promotion if this plan were adopted. Now, we don't object to the attachment of Dental chairs to Medical schools, nor to the promotion of the above described persons. Such arrangement might be of use to the Medical practitioner, and I have no doubt a majority of the Medical profession feel the want of it. But we do object to any such substitution, and utterly oppose a reliance on Medical schools for Dental education. Imagine two or three hundred students hearing four or six lectures a day, *one* of these on some department of Dental science. Day after day each student devotes one-sixth of his time to Dental study, or, perhaps, only a few of them pay any attention to it at all.

Finally, commencement day arrives, and the M. D. is bestowed with a lavish hand. Now, if those graduates are qualified to practice Dental Surgery, when did they study, and how did they become familiar with Dental manipulations? Did that *one* professor, of gigantic intellect and universal genius, infuse it all into them by his *one* lecture a day? And if some are qualified, how are they to be recognized when all are branded with the same significant (?) initials—when all have the same "*ear*-marks?"

The course of studies in our Dental schools, is such as

Dental Surgery requires, and the student, who devotes his mind to Dental science, has not time to study everything else. Besides it is unjust to require twice as much of the Dental, as of the Medical or Theological student. It would be folly to bind a boy seven years to a ship builder, that he might learn to make wagons; and it is just as wise to commence a course of Dental instruction by teaching obstetrics.

The advantages to be gained by associations, for natural improvement, should not be overlooked. The principal object to be sought in association, is improvement. Each member should consider his small stock of ideas as a part of the common fund, from which he may draw, according to his necessities and desires. The man who makes free use of the suggestions of his brethren, at the same time hedging up his own by secrecy or caveats, is quite as honorable as the man who fences in his wood-lands, and turns his hogs in the commons, to eat his neighbors' mast. Indeed, it is quite evident that this spirit of selfishness does much to retard the progress of our profession. We must distinguish ourselves from the charlatan, or we lose not only the confidence of an intelligent public, but our self-respect also. It will not suffice that we cultivate our mental faculties—that we make greater professional attainments than the quack, but our professional conduct must differ from his.

Another cause, which cripples our energies, and destroys our influence, is the prevalence of professional quarrels. These arise, in general, among the self-important, narrow-minded, jealous-hearted members of the profession. Two professional (?) characters run counter to each other's wishes; a paper war commences, and our journals are flooded with their venom. In general, they forget that they are members of civil society, and really forget their right to be so considered. They push on the battle with all the energy of "Kilkenny cats," and the result is often similar to that of the Nova Scotia bull-fight—the one who pushes the other "off the bridge," throws himself so far back on his dignity that he falls off on the opposite side. The combatants generally lose the respect and sympathy, both of their brethren and the public. Each one, however, is goaded on by his clique (whose puffs he mistakes for fame,) little dreaming of the want of interest felt in his cause, by nearly all the profession. It is time these quarrels were treated with the contempt they deserve, and I am pleased to find some of our journals excluding much of this slang from their columns, while others, who do tolerate it, add a few "extra pages," showing plainly that they consider their patrons entitled to better material.

The mass of mankind are incapable of properly estimating professional talent, and the quack thus receives a share of their patronage. Any course which would enlighten the public mind, would, therefore, be calculated to elevate the profession. This might be effected, to some extent, by various methods, but I will not, at present, offer any suggestions on this point.

It is important also, that those entering the profession be qualified for its duties. The position of our society, regulating the reception of students, is a good one and should be strictly adhered to. Those "very fast" men do much to lower the standard of Dental science. A man who has spent ten or fifteen months with a preceptor, finding he can melt gold and extract teeth, fancies himself capable of training a student in half the time. *Fast* men should be discountenanced.

Many other points might be noticed with profit by one more competent, but we will desist, hoping that our past progress will be eclipsed by the future, and that our Association may extends its influence, and live in the confidence of the profession, when its present members shall sleep in the dust.

